# Astrocyte response to IFN-γ limits IL-6-mediated microglia activation and progressive autoimmune encephalomyelitis

**DOI:** 10.1186/s12974-015-0293-9

**Published:** 2015-04-22

**Authors:** Carine Savarin, David R Hinton, Alice Valentin-Torres, Zhihong Chen, Bruce D Trapp, Cornelia C Bergmann, Stephen A Stohlman

**Affiliations:** Department of Neurosciences NC-30, Lerner Research Institute, The Cleveland Clinic, 9500 Euclid Avenue, Cleveland, OH 44195 USA; Department of Pathology, Keck School of Medicine, University of Southern California, Los Angeles, CA 90033 USA

**Keywords:** Progressive multiple sclerosis, Experimental autoimmune encephalomyelitis, Astrocytes, Interferon-γ, Interleukin 6

## Abstract

**Background:**

Therapeutic modalities effective in patients with progressive forms of multiple sclerosis (MS) are limited. In a murine model of progressive MS, the sustained disability during the chronic phase of experimental autoimmune encephalomyelitis (EAE) correlated with elevated expression of interleukin (IL)-6, a cytokine with pleiotropic functions and therapeutic target for non-central nervous system (CNS) autoimmune disease. Sustained IL-6 expression in astrocytes restricted to areas of demyelination suggested that IL-6 plays a major role in disease progression during chronic EAE.

**Methods:**

A progressive form of EAE was induced using transgenic mice expressing a dominant negative interferon-γ (IFN-γ) receptor alpha chain under control of human glial fibrillary acidic protein (GFAP) promoter (GFAPγR1Δ mice). The role of IL-6 in regulating progressive CNS autoimmunity was assessed by treating GFAPγR1Δ mice with anti-IL-6 neutralizing antibody during chronic EAE.

**Results:**

IL-6 neutralization restricted disease progression and decreased disability, myelin loss, and axonal damage without affecting astrogliosis. IL-6 blockade reduced CNS inflammation by limiting inflammatory cell proliferation; however, the relative frequencies of CNS leukocyte infiltrates, including the Th1, Th17, and Treg CD4 T cell subsets, were not altered. IL-6 blockade rather limited the activation and proliferation of microglia, which correlated with higher expression of Galectin-1, a regulator of microglia activation expressed by astrocytes.

**Conclusions:**

These data demonstrate that astrocyte-derived IL-6 is a key mediator of progressive disease and support IL-6 blockade as a viable intervention strategy to combat progressive MS.

## Background

Multiple sclerosis (MS) is characterized by focal inflammation within the central nervous system (CNS), demyelination, axonal damage, and neurological disability [1]. The precise basis for tissue damage associated with MS is unclear. However, experimental autoimmune encephalomyelitis (EAE), a rodent model with numerous characteristics in common with MS, has been essential in understanding the mechanisms underlying MS pathogenesis and has facilitated the identification of therapeutic approaches [2,3]. EAE is mediated by self-reactive T cells directed toward components of myelin [4] secreting interleukin (IL)-17, interferon-γ (IFN-γ), and granulocyte-macrophage colony-stimulating factor [5,6]. Effective therapeutic modalities are currently available for the prevalent relapsing and remitting form of MS. By contrast, no effective therapies have been identified for the treatment of the chronic progressive forms of MS [7]. Indeed, most of the current EAE models provide insight into the acute onset and the relapsing remitting disease course, without the ability to mimic the irreversible progressive disability observed in progressive MS [3,8]. We recently developed a murine model of progressive MS using GFAPγR1Δ transgenic mice in which astrocytes are deficient in IFN-γ signaling characterized by prolonged disability and increased mortality [9]. Several pathological features of progressive MS, including increased demyelination, axonal loss, and astrogliosis, were observed in GFAPγR1Δ mice with chronic EAE [9]. Moreover, the progressive disability and sustained demyelination correlated with elevated expression of IL-6, a cytokine with pleiotropic functions and a therapeutic target for non-CNS autoimmune diseases, such as rheumatoid arthritis [10]. Inhibition of IL-6 signaling is also effective in several murine models of inflammatory diseases, including colitis, asthma, and cancer [11-13].

IL-6, an activator of acute phase responses, is expressed during all forms of inflammation [14], including within the CNS of MS patients [15] and animals with acute EAE [16]. IL-6 is crucial in the induction of CNS autoimmune attack by promoting peripheral induction of self-reactive T cells, facilitating their recruitment into the CNS and limiting the regulatory T cell (Treg) response. Mice lacking IL-6 are resistant to EAE [17-19], consistent with the absence of inflammatory cells within the CNS parenchyma [18,19] and its essential role in T cell activation [20]. IL-6 also interacts with CNS endothelial cells resulting in increased vascular cell adhesion molecule-1 (VCAM-1) expression, a prerequisite for CNS entry of integrin α4β1^+^ effector T cells [21]. In addition to facilitating CNS entry of autoimmune T cells, IL-6 is required for activation of encephalitogenic Th17 cells [22] and enhances acute inflammation by suppressing expression of the Foxp3 transcription factor [23], thereby limiting the suppressive activity of Treg. In addition to a pro-inflammatory role in the context of acute inflammation, IL-6 also exhibits a variety of functions within the CNS either totally or partially independent of its role in inflammation. For example, it is associated with protective CNS functions by promoting neuronal survival and regeneration [24,25], as well as inducing oligodendrocyte differentiation, thereby facilitating myelin repair [26].

Astrocytes are the primary cells secreting IL-6 in the CNS of both patients with MS and in rodents with acute EAE [27,28]. Targeted inhibition of NF-κB in astrocytes is associated with EAE recovery by reducing inflammation and increasing both IL-6 expression [29] and remyelination [30]. These data suggest a protective role for IL-6 during CNS autoimmunity. Nevertheless, constitutive IL-6 expression by astrocytes results in neuronal damage and astrogliosis [31]. Similarly, inhibition of IFN-γ signaling to astrocytes results in a progressive form of EAE and increased IL-6 [9], supporting a deleterious function of IL-6 during progressive disease. Therefore, the role of IL-6 during the remission stages of CNS autoimmunity or during progressive disease remains unclear.

Astrocytes, in addition to secreting IL-6 during both MS and acute EAE, also actively regulate microglia at multiple levels during both pro-inflammatory injury and repair. Astrocytes attenuate microglial activation, reduce microglial secretion of both pro- and anti-inflammatory cytokines, as well as limit microglial-supported T cell proliferation *in vitro* [32]. Specific gene deletions in astrocytes confirm they both attenuate and activate microglia *in vivo* during EAE. For example, Galectin-1 secretion by astrocytes limits microglial activation and clinical symptoms [33]. Similarly, expression of a dominant negative NF-κB repressor in astrocytes diminishes clinical EAE associated with attenuated tumor necrosis factor (TNF) secretion by microglia [29]. By contrast, inhibiting astrocyte IFN-γ signaling sustains disability and correlates with increased TNF and IL-6 [9]. Thus, astrocytes, which play critical roles in both acute and chronic CNS injury, respond to changes by actively shaping the environment and influence repair by altering the destructive or protective state of microglia.

The role of IL-6 in regulating progressive CNS autoimmunity was examined in the transgenic GFAPγR1Δ mouse model of progressive EAE [34]. Similar to acute EAE, neither the inability of the astrocytes to respond to IFN-γ nor the progressive clinical phenotype altered the predominant expression of IL-6 by astrocytes. Blocking IL-6 activity limited progression of clinical symptoms, promoted clinical recovery, and restrained the extent of demyelination and axonal damage. However, neither the composition of leukocyte population nor their predominant cytokine secretion patterns were altered. IL-6 neutralization reduced the frequency of both activated macrophages and microglia, but selectively decreased the sustained proliferation of microglia associated with progressive disease, suggesting a differential effect on microglia versus macrophages. These data indicate that sustained IL-6 secretion by astrocytes during progressive disease is deleterious to both clinical disability and tissue damage and that these affects may be mediated indirectly via microglia. IL-6 blockade may thus provide a viable intervention approach in secondary progressive or primary progressive MS, for which few therapeutic modalities are currently available.

## Materials and methods

### Mice

Homozygous H-2^b^ GFAP/IFN-γR1ΔIC (GFAPγR1Δ) transgenic mice [34], expressing a dominant negative IFN-γ receptor alpha chain under control of human glial fibrillary acidic protein (GFAP) promoter, were bred locally. C57BL/6 (H-2^b^) wild-type (WT) mice were purchased from the National Cancer Institute (Frederick, MD, USA). All procedures were performed in compliance with protocol number 1165 approved by the Cleveland Clinic Institutional Animal Care and Use Committee.

### Experimental autoimmune encephalomyelitis

EAE was induced by subcutaneous injection of 300 μg myelin oligodendrocyte glycoprotein (MOG)^35–55^ peptide emulsified in phosphate-buffered saline (PBS) and incomplete Freund’s adjuvant (IFA; Sigma-Aldrich, St. Louis, MO, USA) supplemented with 5 mg/ml *Mycobacterium tuberculosis*, strain H37Ra (Difco, Detroit, MI, USA), as previously described [9]. Mice were immunized with 200 μl emulsion distributed subcutaneously over two flank sites. One hundred nanograms of pertussis toxin (Sigma-Aldrich) was injected intraperitoneally (i.p.) on the day of initial immunization and 48 h later. Animals were scored daily in a blinded fashion for clinical symptoms as follows: 0 = no signs of disease; 1 = flaccid tail or hind limb weakness; 2 = flaccid tail and hind limb weakness, loss of righting reflex; 3 = partial hind limb paralysis; 4 = complete hind limb paralysis; 5 = moribund or dead.

### **Anti-IL**-**6 treatment**

The MP5-20 F3 hybridoma secreting a rat IgG2a IL-6 neutralizing monoclonal antibody (mAb) and the control GL-113 hybridoma secreting a rat IgG1 mAb specific for β-galactosidase (β-gal) were originally obtained from Dr. Robert Coffman (DNAX Corp, Palo Alto, CA, USA). Hybridomas were adapted to BD Cell Serum-Free Medium (BD, Bedford MA, USA) and the serum-free mAb collected after propagation of the hybridoma in a BD CELLine device. Ig concentrations were determined by optical density at 480 nM, diluted to 1 mg/ml in endotoxin-free PBS, and stored at −20°C until use. Mice were divided into two groups of equivalent average clinical scores and injected i.p. with 500 μg mAb on the specified days post MOG^35–55^ peptide immunization.

### Isolation of CNS-derived cells and flow cytometry

Mice were perfused with ice-cold PBS before the brain and spinal cord were harvested and homogenized in Dulbecco’s PBS using Tenbroeck tissue grinders (Kimble Chase, Vineland, NJ, USA). Mononuclear cells were concentrated from the homogenates by centrifugation at 450 × *g* for 7 min at 4°C. Cell pellets were resuspended in RPMI 1640 medium supplemented with 25 mM HEPES (pH 7.2) and adjusted to 30% Percoll (Pharmacia, Uppsala, Sweden). A 70% Percoll underlay was added prior to centrifugation at 800 × *g* for 30 min at 4°C. Cells were recovered from the 30%/70% interface, washed with RPMI, and then incubated for 10 min on ice in fluorescence activated cell sorting (FACS) buffer with mouse serum and anti-CD16/CD32 mAb (clone 2.4G2, BD Biosciences, San Diego, CA, USA) to limit unspecific binding. FITC-, PE-, PerCP-, and APC-conjugated surface markers (all from BD Biosciences unless specified), including CD45 (30-F11), CD4 (GK1.5), CD11b (clone m1/70), I-A/I-E (clone 2G9), and F4/80 (Serotec, Raleigh, NC, USA), were then added and cells incubated for 30 min on ice. Cells were washed with FACS buffer prior to analysis. For intracellular staining, CNS-derived cells were stimulated for 6 h with phorbol 12-myristate 13-acetate (PMA) (10 ng/ml) (Acros Organics, Geel, Belgium) and ionomycin (1 μM) (Calbiochem, Spring Valley, CA, USA), with Monensin (2 μM) (Calbiochem) added for the last 2 h. Following stimulation, surface molecules were detected as described above. Cells were permeabilized using Cytofix/Cytoperm solution (BD Biosciences) and incubated for 30 min on ice with fluorescent mAb specific for IFN-γ (XMG1.2; BD Biosciences), IL-17 (TC11-18H10; BD Biosciences), or Foxp3 (FJK-16 s; eBiosciences). Cells were then washed using Perm/Wash buffer according to the manufacturer’s instructions. For proliferation, 1 mg of bromodeoxyuridine (BrdU) (BD Biosciences) in PBS was administrated i.p. 24 h prior to sacrifice. Mononuclear cells were prepared from the CNS as described above, stained for surface molecules, and subsequently stained for intranuclear BrdU according to the manufacturer’s instructions using the FITC BrdU flow kit (BD Biosciences). Data were acquired on a FACSCalibur flow cytometer (BD Biosciences) and analyzed using FlowJo software (TreeStar Inc., Ashland, OR, USA).

### Real-time PCR

Following PBS perfusion, snap-frozen brains were placed into TRIzol (Invitrogen, Grand Island, NY, USA) and homogenized using a TissueLyser with stainless beads (Qiagen, Valencia, CA, USA). RNA extraction was performed according to the manufacturer’s instructions followed by DNase I (Ambion, Austin, TX, USA) treatment for 30 min at 37°C. cDNA was then synthetized using M-MLV Reverse Transcriptase (Invitrogen), oligo-dT primers (20 μM) (Promega, Madison, WI, USA), and random primers (20 μM) (Promega). Gene expression analysis was performed by quantitative real-time PCR using a 7500 Fast real-time PCR system (Applied Biosystems, Foster City, CA, USA), SYBR Green Master Mix (Applied Biosystems), and the following primers: GAPDH: F: 5′-TGCACCACCAACTGCTTAG-3′, R: 5′-GGATGCAGGGATGATGTTC-3′; GFAP: F: 5′- CAGTGTGTCAGCCCCACTGA-3′, R: 5′- CAGTGTGTCAGCCCCACTGA-3′. Galectin-1 and heme oxygenase-1 mRNA levels were analyzed using TaqMan primers and 2X Universal TaqMan Fast Master Mix (Applied Biosystems). Transcript levels were normalized to the housekeeping gene GAPDH and converted to a linearized value using the following formula: ${2}^{\left({C_{\mathrm{T}}}^{\mathrm{GAPDH}} - {C_{\mathrm{T}}}^{\mathrm{Gene}}\right)}\times 1,000$, where *C*_T_ represents the threshold cycle value.

### Histopathology

Mice were perfused with ice-cold PBS and spinal cords fixed in Zn Formalin. Spinal cords were separated into six sections, two of each corresponding to the cervical, thoracic, and lumbar regions and embedded in paraffin. Cross sections (6 μm) were stained with hematoxylin and eosin (H&E) or luxol fast blue (LFB). For identification of activated astrocytes and axonal damage, sections were incubated with mouse anti-GFAP (AbCam, Cambridge, MA, USA), SMI-31/SMI-32 (Stenberger Monoclonals Inc., Lutherville, MD, USA), or anti-amyloid precursor protein (APP) mAb (Millipore, Billerica, MA, USA) overnight at 4°C. Biotinylated goat anti-mouse Ab was added for 1 h at room temperature (RT). Sections were then incubated with Vectastain ABC kit (Vector Laboratories, Burlingame, CA, USA) and 3,3′-diaminobenzidine (Sigma-Aldrich). For IL-6 detection, heat-mediated antigen retrieval was performed in 0.1 M citrate buffer, pH 6.0. Sections were then incubated in 0.3% H_2_O_2_ for 20 min followed by rabbit anti-IL-6 Ab (AbCam) overnight at RT. Biotinylated anti-rabbit Ab was added for 30 min at RT. Staining was revealed with Vectastain ABC kit and 3,3′-diaminobenzidine. Stained tissue sections of all six levels on individual glass slides were scanned with an Aperio ScanScope (Aperio, Vista, CA, USA) at 40× and digitally imaged at high resolution. Aperio software was used to quantify areas of demyelination. Axonal damage was quantitated by counting the number of APP^+^ axons in lesions at all six levels and normalized to 100 μm^2^ of demyelinated lesion area.

### Confocal microscopy

Mice were anesthetized and perfused with 4% paraformaldehyde (PFA) in PBS. Spinal cords were removed, separated into portions as described above, and post-fixed in 4% PFA for additional 24 h. Tissues were cryo-protected in 20% glycerol for 24 h before 30-μm free-floating sections were prepared with a sliding microtome (Leica Microsystems, Wetzlar, Germany). Double immunolabeling was performed as previously described [35]. Briefly, sections were microwaved in 0.01 M citrate buffer (pH 6.0) followed by pretreatment with 1% Triton X in PBS. Sections were blocked with PBS containing 3% normal goat serum and 0.01% Triton X for 30 min and incubated with primary Ab diluted in blocking reagent overnight at 4°C (rabbit anti-GFAP, Dako, Carpenteria, CA, USA, 1:1,000; mouse anti-Iba-1, CCF Hybridoma Core, Cleveland, OH, USA, 1:250; goat anti-IL-6, R&D Systems, Minneapolis, MN, USA, 1:20). To confirm specificity, primary Abs were omitted on adjacent sections. The sections were washed and incubated with species-specific secondary Abs conjugated to FITC or Cy5 (Jackson ImmunoResearch, West Grove, PA, USA) for 2 h at RT. Sections were rinsed, mounted with VECTASHIELD (Vector Labs, Burlingame, CA, USA) and examined on a Leica TCS confocal microscope (Leica Microsystems). Images were analyzed offline with Volocity software version 6.1.2 (PerkinElmer, Waltham, MA, USA).

### Statistical analysis

Data represent the mean ± SEM and significance was determined by two-tailed Student’s *t* test or ANOVA with Bonferroni post-test. A value of *P* < 0.05 was considered statistically significant. Graphs were plotted using GraphPad Prism 4.0c software.

## Results

### Astrocytes secrete IL-6 during progressive EAE

**Figure 1:**
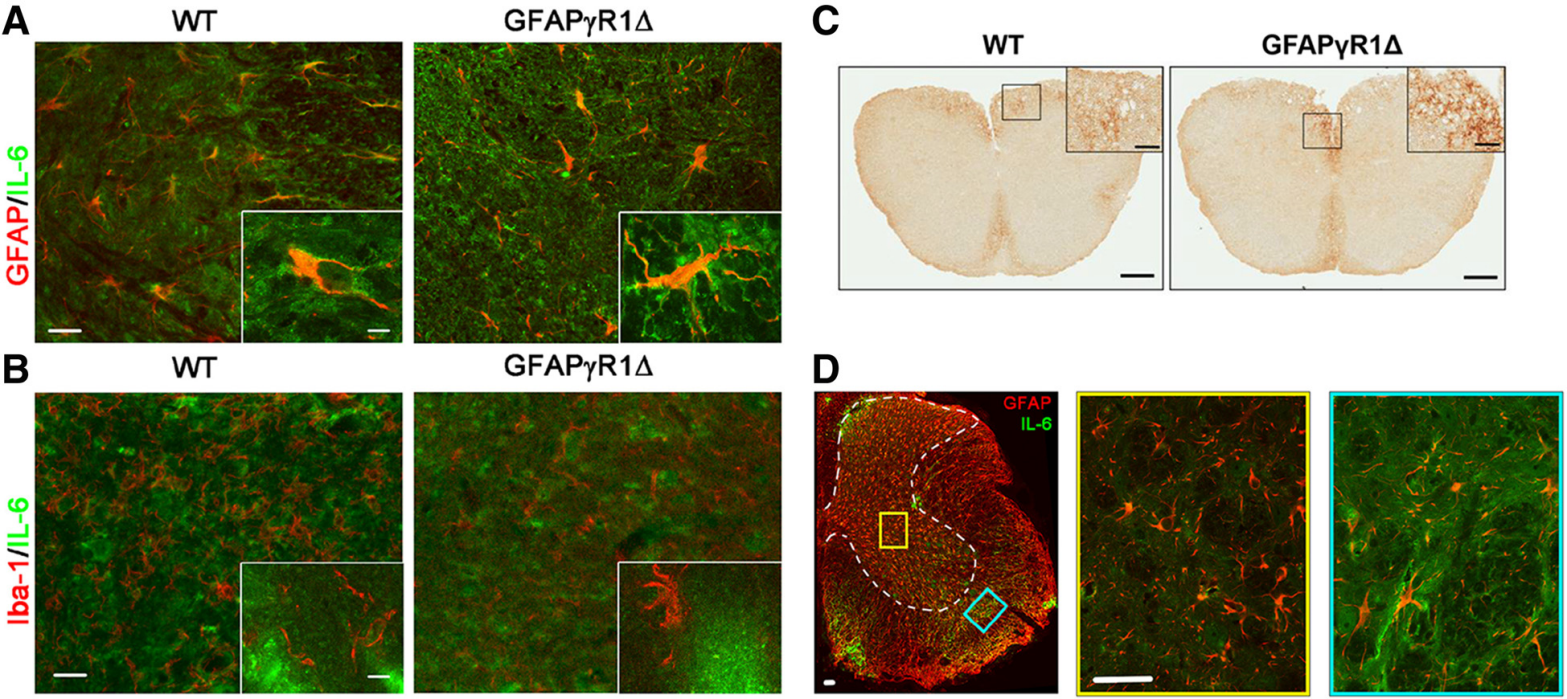
Astrocytes produce IL-6 during chronic EAE. IL-6 co-localizes with GFAP^+^ astrocytes **(A)** in both WT and GFAPγR1Δ mice during the acute EAE, but not with Iba-1^+^ macrophage/microglia **(B)**. Bars = 25 μm in the main panel, 5 μm in the insets. **(C)** During resolving (WT mice) and progressive (GFAPγR1Δ mice) EAE at day 35 post immunization, IL-6-secreting cells were restricted to white matter areas within the spinal cord of both WT and GFAPγR1Δ mice. Bar = 200 μm in the main figure, bar = 50 μm in the inset. **(D)** During progressive EAE, activated astrocytes are found in both gray matter (marked with a dashed line) and white matter lesion areas. No astrocytes producing IL-6 were detected in the gray matter (yellow frame). Only astrocytes in or around white matter lesions produced IL-6 (cyan frame). Images were obtained from the lumbar segments of both WT and GFAPγR1Δ mice and are representative of three mice per group. Bar = 50 μm.

Astrocytes are the predominant source of IL-6 in the CNS of patients with MS and mice with acute EAE [27], and during acute EAE, its expression is independent of the ability of astrocytes to respond to IFN-γ [9]. By contrast, progressive EAE in GFAPγR1Δ mice, in which the astrocytes are unable to respond to IFN-γ, correlated with sustained IL-6 mRNA expression [9]. Its adverse role within the CNS [36] suggested that IL-6 may contribute to progressive disease. As numerous cell types including myeloid cells and microglia can secrete IL-6, we determined if the inability of astrocytes to respond to IFN-γ signaling altered the cellular source or distribution of IL-6 during acute and chronic/progressive disease. The majority of cells secreting IL-6 during acute EAE in both WT and GFAPγR1Δ mice co-express GFAP, confirming their identity as astrocytes (Figure 1A). By contrast, during acute EAE in both groups, IL-6 expression was undetectable in Iba-1^+^ cells (Figure 1B). These data demonstrate that the predominant source of IL-6 during acute EAE is not altered by the inability of astrocytes to respond to IFN-γ. Neither the cell type(s) secreting IL-6 nor their anatomical location relative to the white matter lesions has been established during either chronic or progressive forms of EAE. In contrast to WT mice, where astrogliosis is diminished during chronic EAE and limited to white matter areas, astrocyte activation is sustained during progressive disease in GFAPγR1Δ mice, including in gray matter distal from the demyelinating lesions [9]. Although the number of cells secreting IL-6 in the absence of IFN-γ signaling to astrocytes was increased relative to WT mice, IL-6^+^ cells in mice with progressive EAE were located within white matter areas (Figure 1C), similar to the distribution in WT mice. Moreover, IL-6 remained undetectable in Iba-1^+^ cells (data not shown) and co-localized mainly with GFAP^+^ cells located within or adjacent to areas of myelin loss (Figure 1D), supporting astrocytes as the primary source of IL-6 during both acute and chronic EAE. Although IFN-γ signaling limits astrocyte activation to areas of demyelination, IL-6 secretion is independent of sustained activation as only astrocytes associated with the areas of myelin loss secreted IL-6 during chronic EAE.

### IL-6 limits clinical improvement

**Figure 2:**
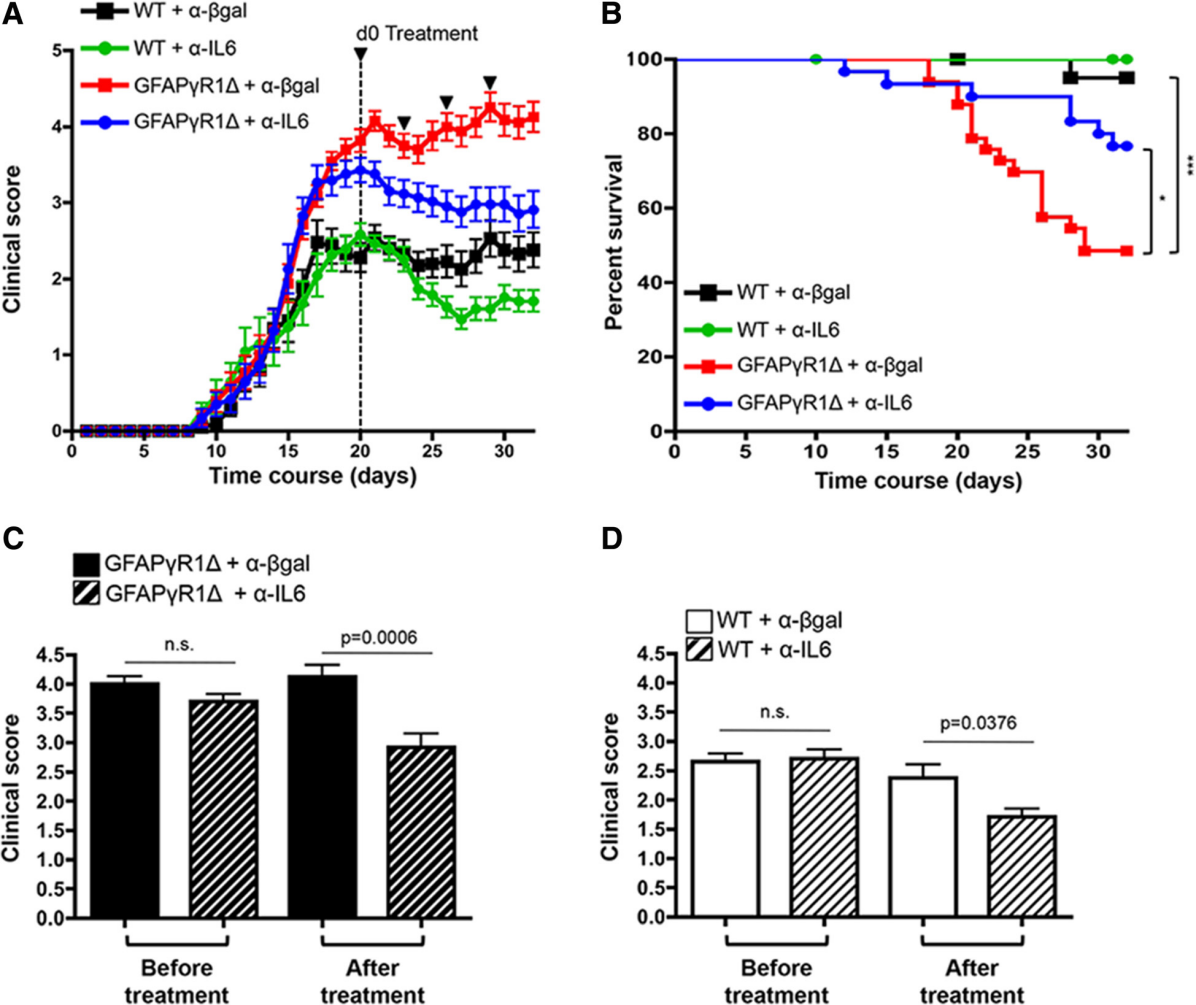
Anti-IL-6 treatment ameliorates clinical disease. GFAPγR1Δ and WT mice were separated into two groups with identical clinical scores at the peak of EAE and received either the isotype control α-β-gal or α-IL-6 mAb treatment. **(A)** Disease progression was inhibited in GFAPγR1Δ mice and recovery was promoted in WT mice by anti-IL-6 treatment. Arrowheads represent time of injection. **(B)** Minimal mortality in WT mice treated with α-β-gal or α-IL-6 mAb. Survival decreased in GFAPγR1Δ mice treated with isotype control mAb. By contrast, mortality was reduced by treatment of GFAPγR1Δ mice with α-IL-6 mAb. Kaplan-Meier survival curves with *P < 0.05, ***P < 0.001, log-rank test. **(C, D)** At day 32 post immunization (12 days after initial α-IL-6 treatment), both GFAPγR1Δ **(C)** and WT **(D)** mice treated with α-IL-6 exhibited improved clinical disease. Statistical differences determined by a two-tailed unpaired *t* test. Data represent the average ± SEM with GFAPγR1Δ + α-β-gal (*n* = 33), GFAPγR1Δ + α-IL-6 (*n* = 30), WT+ α-β-gal (*n* = 20), and WT+ α-IL-6 (*n* = 20) from at least three separate experiments.

IL-6 has been implicated in the pathogenesis of MS, and its inhibition is efficacious in treating human peripheral autoimmune diseases [37]. Moreover, IL-6 blockade prior to, or concomitant with, induction of EAE eliminated both clinical disease and CNS inflammation [17,38]. Although IL-6 blockade after initial EAE clinical symptoms appeared was ineffective [38], the contribution of IL-6 to progressive EAE remains unknown. GFAPγR1Δ mice exhibiting essentially identical clinical symptoms at the peak of EAE were therefore divided into two treatment groups (see Figure 2A,C). One group was treated by intraperitoneal injection of 500 μg neutralizing anti-IL-6 mAb, while the other group received an equal amount of isotype control rat IgG mAb. Following initial treatment, all mice were treated every third day for a total of four injections. Diminution of clinical disease severity was readily apparent after the initial injection of anti-IL-6 mAb (Figure 2A), and a reduction in the progression of clinical symptoms was sustained by IL-6 neutralization (Figure 2A,C). In contrast to the resolving disease in WT mice, sustained clinical disease in GFAPγR1Δ mice is associated with accumulating mortality (Figure 2B). Anti-IL-6 treatment improved survival of GFAPγR1Δ mice during progressive EAE, reducing mortality from 52% to 24% (Figure 2B). To determine if IL-6 neutralization also promoted recovery in non-progressive EAE, an identical experiment was carried out in WT mice. Similar to GFAPγR1Δ mice, mice were divided into two groups at the peak of clinical disease and one group received anti-IL-6 and the other isotype control mAb (Figure 2A,D). Although improvement of symptoms was less than that observed during progressive disease in GFAPγR1Δ mice (Figure 2A), anti-IL-6 treatment of WT mice with EAE also mediated a reduction of approximately 0.7 score units compared to the control group (Figure 2A,D). Thus, these data demonstrate that IL-6 blockade limits mortality and promotes recovery during a paralytic autoimmune disease associated with progressive disability and to a lesser extent during resolving conditions.

### IL-6 enhances tissue damage during progressive EAE

**Figure 3:**
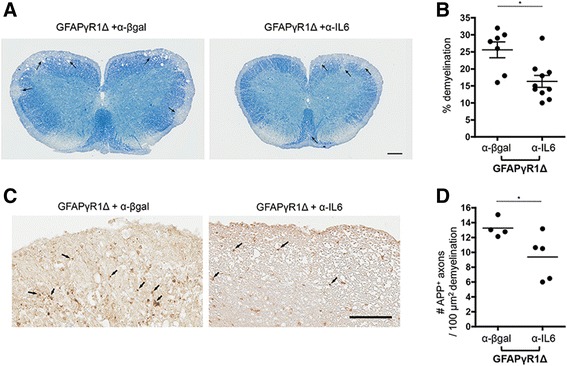
Inhibition of disease progression in GFAPγR1Δ mice correlates with decreased demyelination and axonal damage. Spinal cord sections of GFAPγR1Δ mice treated with α-β-gal and α-IL-6 mAbs at day 32 post immunization stained with LFB **(A)** or anti-APP **(C)** to assess demyelination and axonal damage, respectively. Representative sections from three experiments with three individual mice per group per experiment. In **(A)**, areas of demyelination are shown with arrows. Bar = 200 μm. **(B)** Percentage area of demyelination in spinal cord white matter calculated by analysis of transverse sections at six separate levels per mouse. Data represent the mean ± SEM of seven to nine individual mice per group from two separate experiments. APP^+^-damaged axons **(C)** shown with arrows. Bar = 100 μm. **(D)** Number of APP^+^ axons per 100-μm^2^ area of demyelination analyzed in GFAPγR1Δ mice treated with α-β-gal and α-IL-6 mAb at day 32 post immunization. Data represent the mean ± SEM of four to five individual mice per group from two separate experiments. Statistical differences determined by a two-tailed unpaired *t* test with *P < 0.05.

Demyelination and axonal damage, hallmarks of EAE as well as relapsing remitting and chronic progressive MS [1], contribute to clinical disease. To determine if reduced clinical disease severity during progressive EAE mediated by anti-IL-6 treatment correlated with reduced tissue damage, individual spinal cords from the treated and isotype control mice were analyzed at six levels, 4 days following the last treatment. The extensive demyelination associated with the inability of astrocytes to respond to IFN-γ [9] was diminished by IL-6 neutralization (Figure 3A). The extent of protection averaged over six sections in multiple animals demonstrated approximately 40% reduction in the white matter areas involved in myelin loss (Figure 3B). Axonal damage, a possible contributor to decreased disability [39], was examined by determining APP expression as well as phosphorylated and non-phosphorylated neurofilaments. Both approaches indicated that axonal damage was confined to areas within lesions and correlated with the extent of demyelination. Importantly, IL-6 mAb treatment reduced the extent of not only myelin damage but also axonal damage within the lesions (Figure 3C,D). These data demonstrate that sustained IL-6 secretion by astrocytes enhances clinical disease as well as both the extent of demyelination and its associated axonal damage.

### IL-6-independent astrogliosis

**Figure 4:**
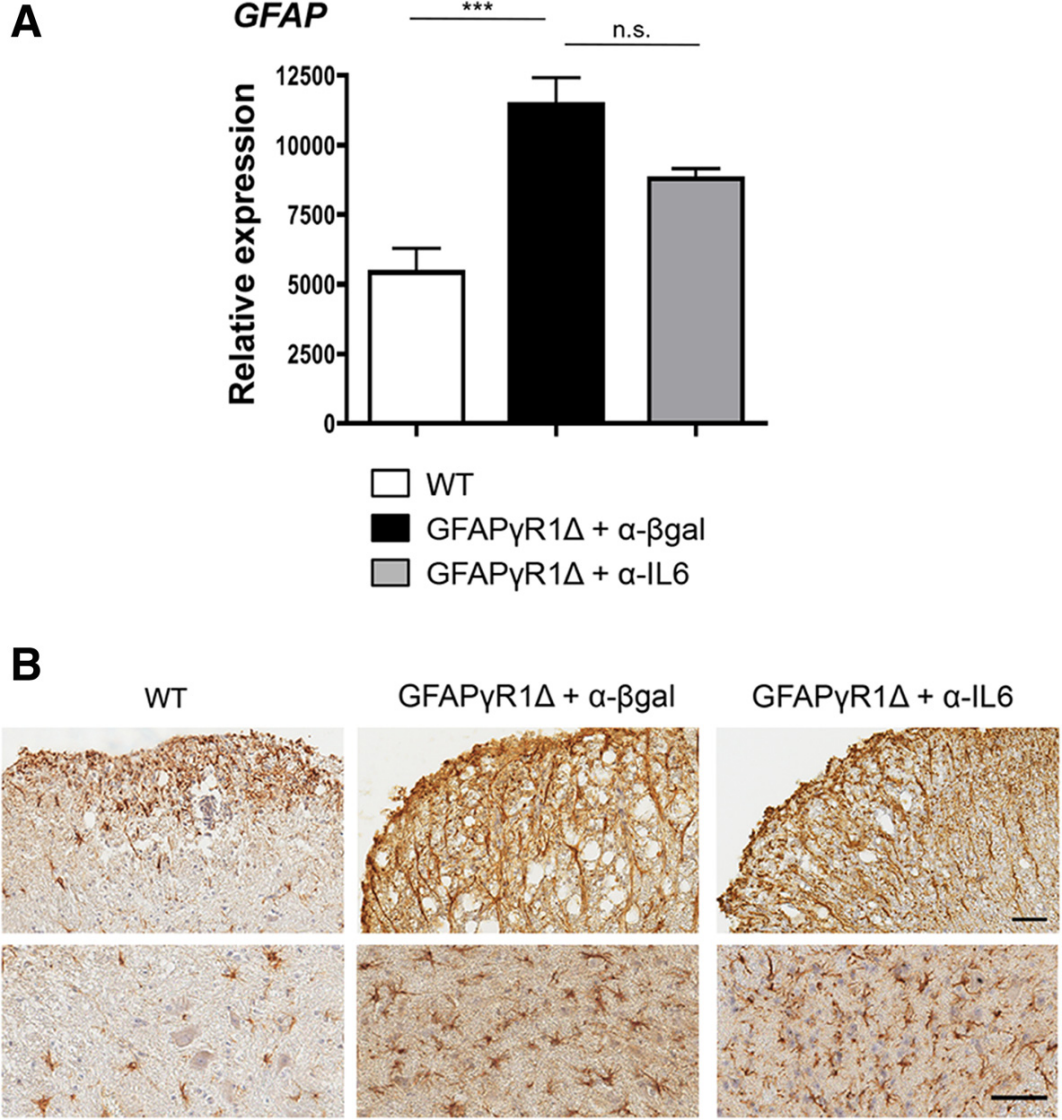
IL-6 blockade has a limited effect on astrocyte activation. **(A)** GFAP mRNA expression analyzed by real-time PCR at day 32 post immunization in the spinal cords of WT, GFAPγR1Δ + α-β-gal, and GFAPγR1Δ + α-IL-6 mice with *n* = 3 to 4 individual mice per group. Statistics were calculated with one-way ANOVA with Bonferroni post-test with ***P < 0.001. **(B)** Immunohistochemical staining for GFAP in WT, GFAPγR1Δ + α-β-gal, and GFAPγR1Δ + α-IL-6 mice. Upper panels show GFAP labeling within areas of white matter demyelination. Lower panels show GFAP labeling in gray matter distal from areas of demyelination. Bar = 50 μm.

Astrogliosis, induced by virtually all perturbations of the CNS environment [40], is also associated with white matter lesions in MS and EAE [41]. Progressive EAE in GFAPγR1Δ mice showed extended astrocyte activation into gray matter areas, including those distal from demyelinating lesions [9]. IL-6 is one of several molecules supporting astrogliosis [42,43]. We therefore considered the possibility that increased IL-6 within the CNS of GFAPγR1Δ mice with progressive EAE accounted for, or contributed to, either the sustained astrogliosis or the unique global distribution of activated astrocytes in the gray matter. Comparative analysis of mRNA encoding GFAP, a marker of astrocyte activation, during progressive EAE confirmed increased astrocyte activation in GFAPγR1Δ mice relative to WT mice (Figure 4A). However, GFAPγR1Δ mice treated with anti-IL-6 exhibited only a minor trend toward decreased GFAP mRNA expression (Figure 4A), suggesting that although IL-6 secretion was sustained in the areas of white matter damage (Figure 1), it did not contribute to the sustained global astrocyte activation. This was confirmed by comparing the distribution of activated astrocytes in the control and anti-IL-6-treated groups. Neither numbers of activated astrocytes within, or adjacent to, demyelinating areas nor those in the gray matter were affected by IL-6 neutralization (Figure 4B). These observations suggest that clinical improvement, diminished demyelination, and limited axonal damage mediated by IL-6 neutralization are independent of overall astrocyte activation.

### IL-6 regulated CNS inflammation

**Figure 5:**
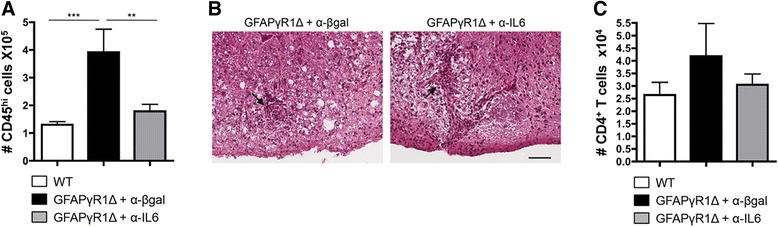
Inflammatory leukocytes during progressive EAE are reduced by IL-6 blockade. **(A)** Total number of bone marrow-derived inflammatory cells (CD45^hi^). Data represent the mean ± SEM of two separate experiments with *n* = 4 per group per experiment. Statistics were calculated with one-way ANOVA with Bonferroni post-test with **P < 0.01; ***P < 0.001. **(B)** Inflammatory cells within the spinal cord sections of GFAPγR1Δ mice treated with α-β-gal or α-IL-6 mAb at day 32 post immunization identified with H&E. Inflammatory cells were limited to lesions and were both perivascular (arrows) and within the parenchyma. Bar = 50 μm. Representative sections from two experiments with four individual mice per group per experiment. **(C)** CD4^+^ T cells (CD45^hi^CD4^+^) within the CNS of WT, GFAPγR1Δ + α-β-gal, and GFAPγR1Δ + α-IL-6 mice analyzed by flow cytometry at day 32 post immunization. Data represent the mean ± SEM of two separate experiments with *n* = 4 per group per experiment.

The contribution of inflammation to the disability associated with MS and EAE is unclear. Nevertheless, astrocytes are critical in limiting the autoimmune inflammatory response [44], and their IL-6 secretion may enhance autoimmune inflammation by preventing T cell apoptosis [11]. To determine if IL-6 influenced CNS inflammation, bone marrow-derived CD45^hi^ inflammatory cells within the CNS were examined by flow cytometry. Increased numbers of inflammatory cells in the CNS of mice with progressive EAE relative to resolving disease confirmed IFN-γ signaling to astrocytes regulates the magnitude of inflammation (Figure 5A). Neutralization of IL-6 reduced the number of CNS inflammatory cells to approximately the same level as in WT mice with resolving EAE (Figure 5A), suggesting that increased inflammation associated with the inability of astrocytes to respond to IFN-γ signaling is due, at least in part, to sustained IL-6 secretion. Despite the correlation of reduced CNS inflammation with clinical improvement, reduced demyelination, reduced axonal damage, and decreased disability, anti-IL-6 treatment did not alter the overall distribution of inflammatory cells, which remained in both perivascular and intra-parenchymal locations within the lesion areas (Figure 5B).

**Figure 6:**
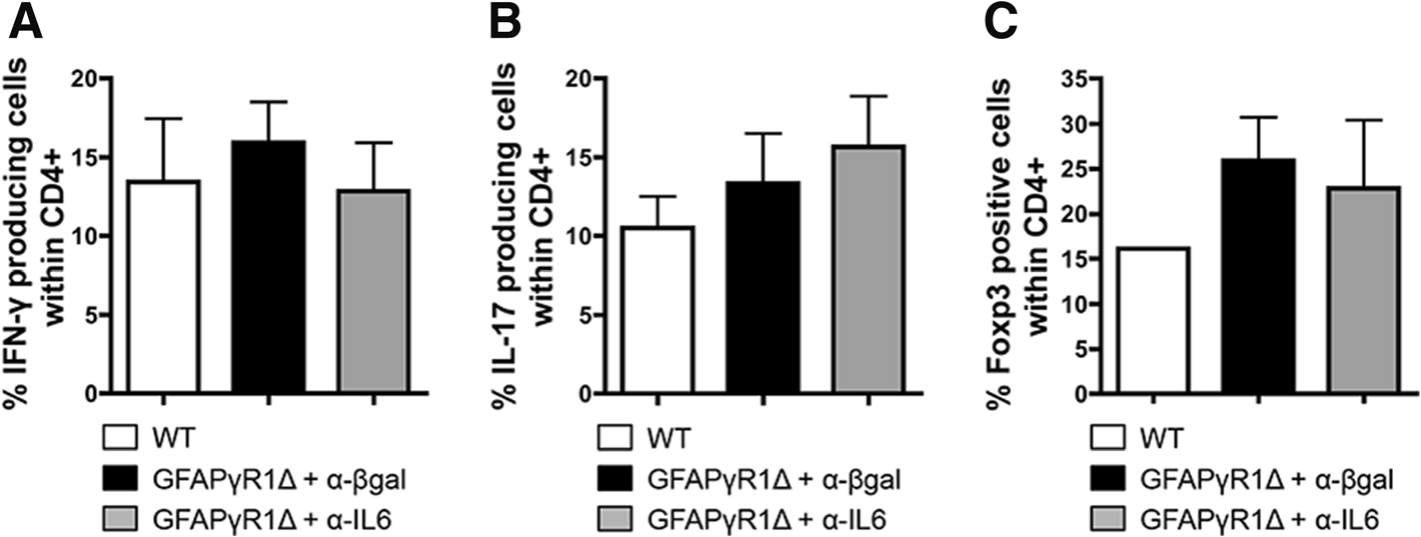
CD4^+^ T cell response is not altered by anti-IL-6 treatment. Frequencies of CD4^+^ T cells within the CNS of WT, GFAPγR1Δ + α-β-gal, and GFAPγR1Δ + α-IL-6 mice producing IFN-γ **(A)** and IL-17 **(B)** as well as cells expressing Foxp3 **(C)** determined by flow cytometry at day 32 post immunization. Data represent the mean ± SEM of two separate experiments with *n* = 4 pooled mice per group per experiment.

IL-6 has a central role in facilitating CNS inflammation while potentially inhibiting Treg function [22,45], but its role in regulating effector T cell populations within the CNS during progressive EAE has not been described. Flow cytometric analysis revealed only minor alterations in total CD4^+^ T cell numbers, with a slight decrease induced by anti-IL-6 treatment (Figure 5C). Whereas IL-6 neutralization had only a limited effect on overall CD4^+^ T cells, it may be protective by modifying the frequency of effector T cells or Treg. The relative frequencies of effector T cells secreting IFN-γ and IL-17, as well as the frequency of Treg, were compared within the CNS of GFAPγR1Δ mice treated with anti-IL-6 or control mAb during progressive EAE, as well as in WT mice with EAE. No differences in the frequency of CD4^+^ T cells secreting IFN-γ (Figure 6A) or IL-17 (Figure 6B) were detected after anti-IL-6 treatment of GFAPγR1Δ mice. In addition, there were no changes in Foxp3^+^ Treg which correlated with anti-IL-6-mediated protection (Figure 6C). Decreased inflammation mediated by IL-6 neutralization is consistent with limiting apoptosis [11]. However, only a limited number of caspase 3^+^ cells were detected in the spinal cords of either GFAPγR1Δ or WT mice at 32 days post immunization (data not shown). Furthermore, no differences in the anatomical location of caspase 3^+^ cells were noted, irrespective of the treatment group, supporting the absence of an IL-6-mediated inhibition of anti-apoptotic protection. These data suggested that neutralization of IL-6 influenced the overall inflammation by decreasing proliferation, rather than facilitating apoptosis or altering the frequency of effector T cells or Treg.

**Figure 7:**
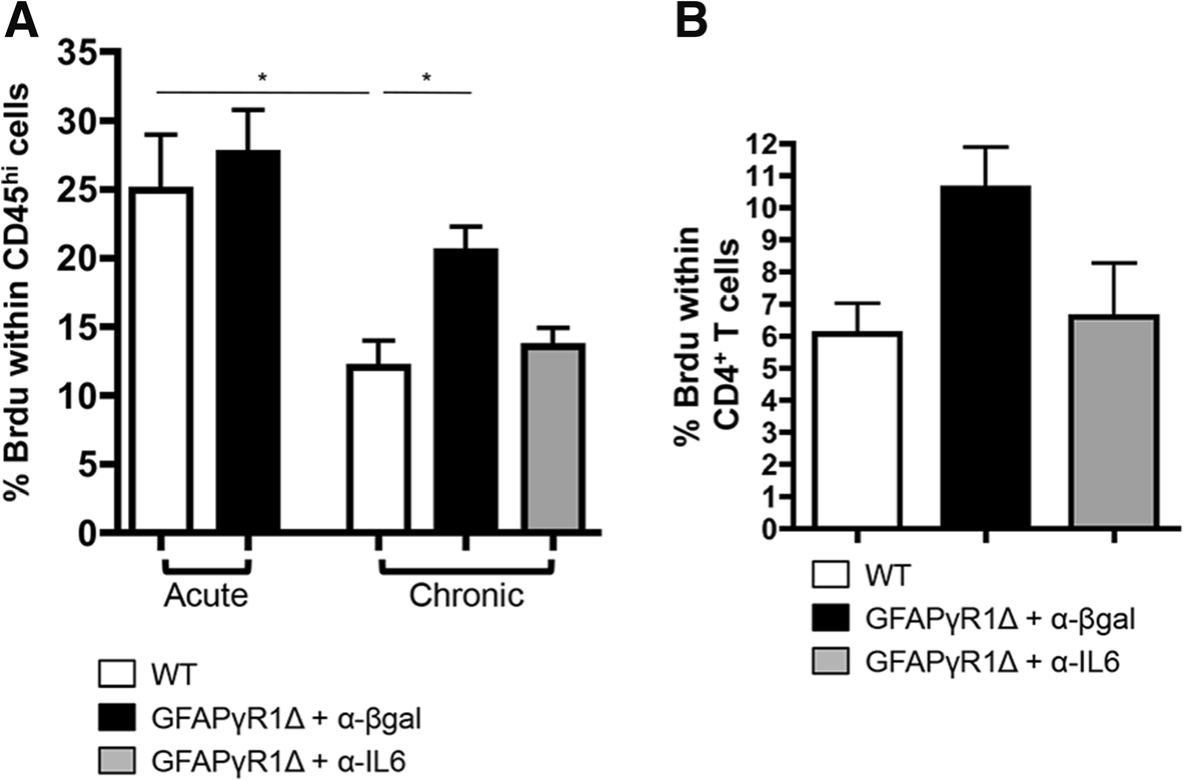
Proliferation of inflammatory cells during progressive EAE is reduced by anti-IL-6 treatment. **(A)** Proliferation of CD45^hi^ cells within the CNS of WT and GFAPγR1Δ mice during acute EAE (day 20 post immunization) or WT, GFAPγR1Δ + α-β-gal, and GFAPγR1Δ + α-IL-6 mice during chronic EAE (day 32 post immunization) measured by BrdU staining and flow cytometry. Four individual mice were analyzed per group per time point. **(B)** Percentage of BrdU-positive cells within CD4^+^ cells analyzed at day 32 post immunization in WT and GFAPγR1Δ mice treated with α-β-gal or α-IL-6 mAb. Data represent the mean ± SEM of two separate experiments with *n* = 4 per group per experiment. Statistics were calculated with one-way ANOVA with Bonferroni post-test with *P < 0.05.

IL-6-dependent proliferation of inflammatory cells was examined via a pulse of BrdU. During acute EAE, numbers of proliferating CNS inflammatory cells were similar in WT and GFAPγR1Δ mice (Figure 7A). By contrast, proliferation of CNS inflammatory cells was sustained during progressive EAE, while it declined in WT mice (Figure 7A). Consistent with a reduction of CNS inflammation in GFAPγR1Δ mice treated with anti-IL-6 mAb, proliferation declined to levels equivalent to those in WT mice during chronic EAE (Figure 7A). Analysis of CD4^+^ T cell proliferation (Figure 7B) confirmed a limited alteration in CD4^+^ T cells (Figure 5C). Although the minimal increase in CD4^+^ T cell proliferation in GFAPγR1Δ mice was reduced by IL-6 neutralization to approximately the level of CD4^+^ T cell proliferation in WT mice, the reduction did not reach statistical significance (Figure 7B). These data suggest that within the CNS, IL-6 facilitates proliferation of inflammatory cells other than CD4^+^ T cells.

### IL-6 regulated microglia activation

**Figure 8:**
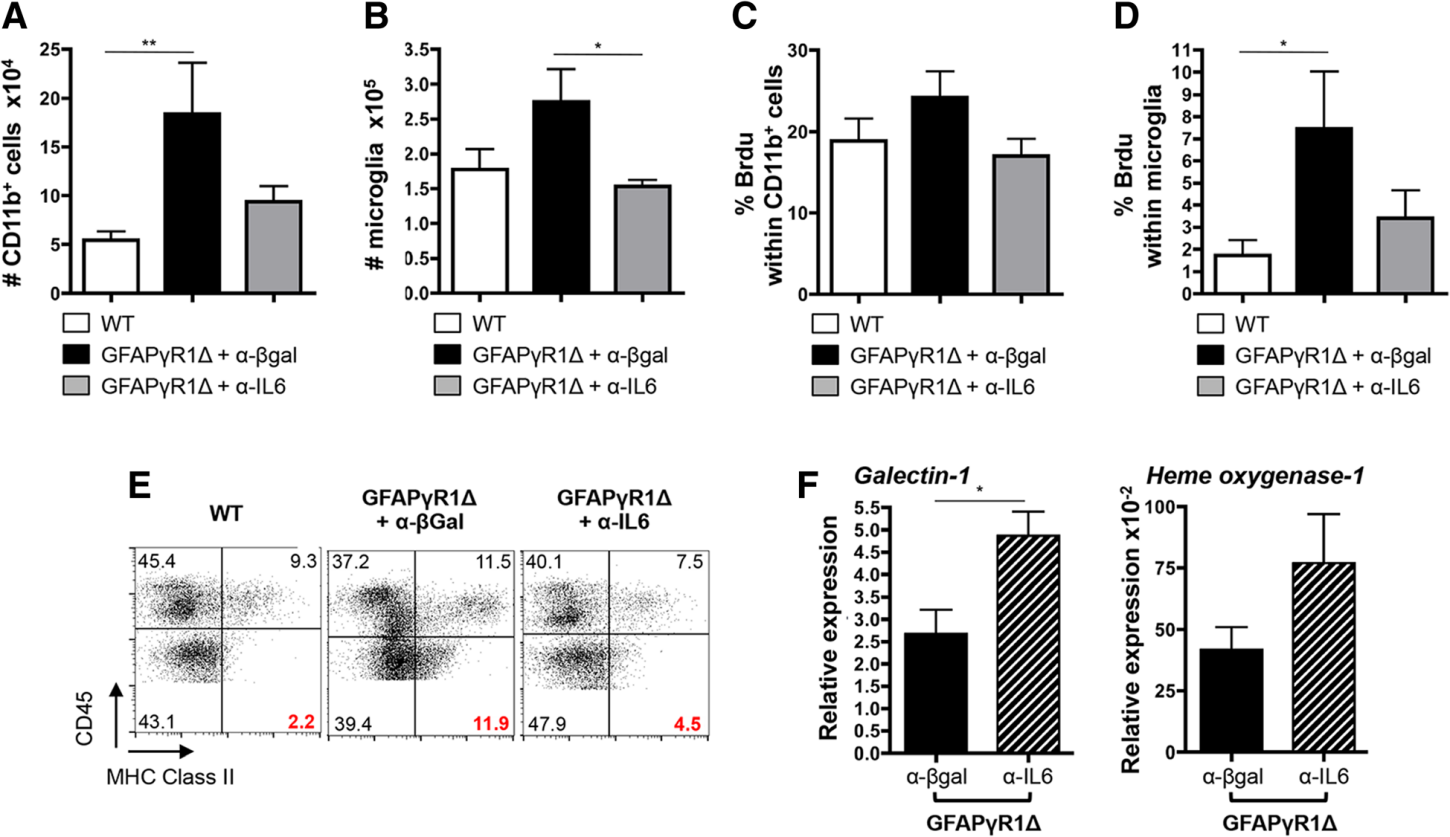
Increased microgliosis characteristic of progressive EAE is decreased after anti-IL-6 treatment. Total number of macrophages (CD45^hi^CD11b^+^) **(A)** and microglia (CD45^lo^CD11b^+^) **(B)** within the CNS of WT and GFAPγR1Δ mice treated with α-IL-6 or α-β-gal mAb at day 32 post immunization. Percentage of BrdU-positive macrophages (CD45^hi^CD11b^+^) **(C)** and microglia (CD45^lo^CD11b^+^) **(D)** in WT and GFAPγR1Δ mice treated with α-β-gal or α-IL-6 analyzed at day 32 post immunization by flow cytometry. Data represent the mean ± SEM of two separate experiments with *n* = 4 per group per experiment. Statistics calculated with one-way ANOVA with Bonferroni post-test with *P < 0.05; **P < 0.01. **(E)** Microglia activation, characterized by MHC class II expression within CD45^lo^CD11b^+^ cells, analyzed at day 32 post immunization by flow cytometry in WT, GFAPγR1Δ + α-β-gal, and GFAPγR1Δ + α-IL-6 mice. **(F)** Galectin-1 and heme oxygenase-1 mRNA expression within the brain of GFAPγR1Δ mice treated with α-β-gal or α-IL-6 at day 32 post immunization. Data represent the mean ± SEM of four individual mice per group. Statistics were calculated with by a two-tailed unpaired *t* test with *P < 0.05.

Recruitment of bone marrow-derived macrophages (BMDM) into the CNS is required for demyelination during acute EAE [46,47]; however, the relative contribution of macrophages and microglia to tissue damage during progressive MS or EAE is unclear. IL-6 stimulates microglia proliferation *in vitro* [48] and microglia activation *in vivo* [43]. Flow cytometric analysis of CD45^hi^ BMDM and CD45^lo^ microglia showed that progressive EAE was associated with an increased number of both BMDM and microglia relative to WT mice, which declined to WT levels following IL-6 neutralization (Figure 8A,B). A pulse of BrdU was employed to determine if the increase in BMDM and microglia in GFAPγR1Δ mice relative to WT mice correlated with increased IL-6-mediated proliferation. BMDM proliferation was similar in all groups during chronic EAE (Figure 8C), and microglia proliferation in WT mice remained at low levels during chronic EAE (Figure 8C). By contrast, microgliosis was increased in GFAPγR1Δ mice during progressive EAE (Figure 8D). Consistent with its therapeutic effects, IL-6 neutralization decreased microglia proliferation to approximately the levels in WT mice (Figure 8D). Sustained proliferation suggested that the inability of astrocytes to respond to IFN-γ correlated with sustained microglial activation. Major histocompatibility complex (MHC) class II expression, as a measure of both activation and potential for antigen presentation, was observed in a higher percentage of both macrophages and microglia derived from GFAPγR1Δ mice with progressive EAE compared to the respective populations derived from WT controls (Figure 8E). The increase in class II-expressing microglia was more dramatic (2% in WT vs. 12% in GFAPγR1Δ mice) compared to the minimal increase in class II-expressing BMDM (9% in WT vs. 12% in GFAPγR1Δ mice). Coupled with increased proliferation, these data suggest that the inability of astrocytes to respond to IFN-γ results in sustained microglial activation, possibly due to a diminished ability of astrocytes to secrete microglial inhibitory molecules, which are ameliorated by IL-6 neutralization. Galectin-1 and heme oxygenase 1 (HO-1) participate in the astrocyte and microglia crosstalk and contribute to the regulation of CNS inflammation [30,33]. Astrocyte-derived Galectin-1 deactivates microglia during EAE [33], whereas increased HO-1 in microglia is regulated by astrocytes [49]. Both Galectin-1 and HO-1 mRNA expression were increased in GFAPγR1Δ mice with progressive EAE receiving anti-IL-6 treatment (Figure 8F), supporting the concept that IFN-γ signaling to astrocytes is essential in limiting IL-6 secretion by astrocytes and reducing microglia activation.

## Discussion

Induction of EAE in mice in which IFN-γ signaling to astrocytes is inhibited results in a progressive form of EAE characterized by sustained paralytic disease, demyelination, axonal damage, and escalating mortality. A hallmark of this progressive disease, distinguishing it from the resolving form, is global astrocyte activation indicated by hypertrophic astrocytes with increased GFAP expression. Although astrogliosis is associated with white matter lesions in both the acute and chronic forms of non-progressive EAE, activation in progressive EAE was more global including astrocytes within gray matter areas [9]. Progressive EAE also correlated with diminished anti-inflammatory cytokines and increased pro-inflammatory cytokines, including IL-6 [9]. IL-6, although undetectable in the naïve CNS, may contribute to physiological processes such as astrogliogenesis and neuronal differentiation [50] and has also been implicated in a variety of chronic CNS diseases including MS, Alzheimer’s, Parkinson’s, and Huntington’s diseases [50]. In both patients with MS and during acute EAE, astrocytes are the major source of IL-6 [15,16], suggesting that the increase in IL-6 resulted from the sustained astrogliosis in the absence of IFN-γ signaling. Nevertheless, IL-6 secretion is limited to astrocytes proximal to areas of demyelination, suggesting an intimate relationship between IL-6 secretion and myelin loss, which led us to investigate the pathogenic role of IL-6 during progressive EAE.

Neutralization of IL-6 during the chronic progressive phase of EAE limited disease progression, which correlated with decreased demyelination and axonal damage, hallmarks of the CNS lesions in patients with relapsing remitting and progressive MS [51]. Nevertheless, decreased tissue damage following IL-6 inhibition did not reduce the extent of astrogliosis, suggesting that IL-6 does not contribute to sustained astrocyte activation and that its protective effect is independent of astrogliosis, at least over the time course of these experiments. Moreover, IL-6 did not increase within the CNS of either anti-IL-6 treated WT or GFAPγR1Δ mice (data not shown), as was observed in mice treated with anti-IL-6 prior to disease onset [52]. IL-6 blockade during progressive EAE correlated with decreased CNS inflammation, suggesting the possibility that IL-6 directly alters the response of self-reactive T cells. Indeed, as a component of peripheral immune activation, IL-6 regulates T cell activation and cytokine secretion, limits Treg-suppressive activity, and alters the endothelial cells associated with the blood–brain barrier, thereby controlling T cell egress into the CNS parenchyma [22,53]. Nevertheless, blocking IL-6 prior to the onset of acute EAE has led to conflicting results. Indeed, IL-6 blockade prevented EAE development by limiting both Th1 and Th17 cell differentiation [16,38,52]. By contrast, Willenborg et al. [54] were unable to detect any effect of anti-IL-6 treatment; IL-6 blockade initiated at disease onset also had little effect on disease development [38], consistent with the concept that disease progression during acute EAE is IL-6 independent once T cells are activated. The present data show that inhibition of IL-6 during progressive EAE reduced inflammation without altering the frequency of either Th17 or Th1 cells, again suggesting a mode of action distinct from IL-6 blockade prior to disease onset [38]. In support of a mechanism independent of autoimmune T cell activation within the CNS, Foxp3^+^ Treg were not increased within the CNS following sustained IL-6 neutralization, although IL-6 is reported to limit Treg activity via suppressing Foxp3 expression [23]. In addition, no effects on apoptotic cell numbers by anti-IL-6 treatment implied protection was independent of IL-6 anti-apoptotic activity [11]. IL-6 also had a limited effect on sustaining CD4 T cell proliferation during progressive EAE. Moreover, the inability of astrocytes to present class II antigen in the context of *in vivo* inflammation [55] suggested an indirect effect of sustained IL-6 secretion on antigen presenting cells in supporting self-reactive T cell activation.

IFN-γ signaling to astrocytes limited the overall inflammatory response within the CNS [9]. The increased CNS inflammation during progressive EAE correlated with increased numbers of BMDM and activated microglia exhibiting sustained MHC class II expression. IL-6 neutralization reduced the number of both activated BMDM and microglia, although IL-6 preferentially increased microglial proliferation. Similarly, MHC class II expression was down regulated by IL-6 blockade more efficiently on microglia. This observation may explain the limited effect of anti-IL-6 treatment on CD4^+^ T cell proliferation, as microglia exhibit limited antigen presentation functions *in vivo* [56,57]. These data suggest that increased tissue damage may correlate with the inability of the astrocyte to down regulate microglial activity in the absence of IFN-γ signaling. Recent *in vivo* data support the concept that astrocytes regulate microglial activity during inflammation. Inhibition of NF-κB in astrocytes diminished clinical disease during EAE by attenuating microglia activation [29,30]. Similarly, Galectin-1 secretion by astrocytes limits both microglial activation and EAE [33], whereas astrocyte-mediated induction of HO-1 in microglia limits neuroinflammation [49]. Increased expression of both Galectin-1 and HO-1 after anti-IL-6 treatment, associated with decreased microglia proliferation and MHC class II expression, thus supports the concept that the crosstalk between astrocytes and microglia during the chronic phase of EAE is essential for disease resolution. However, it remains unclear whether astrocyte-derived IL-6 acts directly on microglia or indirectly via an autocrine-induced alteration in astrocyte function [58]. It is also possible that IL-6 itself alters astrocyte functions, which also contributes to tissue damage during progressive EAE. Astrocytes regulate diverse CNS functions from neurogenesis to defending from both internal and external insults [40]. Their critical contributions to regulating chronic CNS autoimmune disease are supported by the expanding number of alterations in astrocyte function that result in progressive types of EAE [9,59,60]. Furthermore, detrimental effects of IL-6 secretion by astrocytes on CNS function and pathobiology are exemplified by analysis of mice in which IL-6 is constitutively expressed by astrocytes [61]. Sustained IL-6 results in localized neuroinflammation, neurodegeneration, loss of blood–brain barrier integrity, and impaired learning [31,62,63]. Finally, reduced disease severity after elimination of B cells secreting IL-6 in two EAE models as well as in patients with relapsing remitting MS [64,65] supports a destructive role for IL-6 during CNS autoimmunity.

## Conclusions

These data demonstrate that one anti-inflammatory activity of IFN-γ is to prevent sustained IL-6 expression by astrocytes, which plays an essential role in disease progression during progressive EAE. IL-6 is critical in mediating demyelination, axonal damage, and sustaining both CNS inflammation and disability during progressive EAE. These data demonstrated the therapeutic potential of IL-6 blockade in limiting both the disability and tissue damage in a model of progressive EAE. This approach, currently in use to treat patients with peripheral autoimmune disease, may thus be efficacious in the treatment of the progressive forms of MS.

## Abbreviations

APP: amyloid precursor protein
BMDM: bone marrow-derived macrophage
BrdU: bromodeoxyuridine
CNS: central nervous system
EAE: experimental autoimmune encephalomyelitis
β-gal: β-galactosidase
GFAP: glial fibrillary acidic protein
H&E: hematoxylin and eosin
HO-1: heme oxygenase-1
IL: interleukin
IFN-γ: interferon-γ
i.p.: intraperitoneally
LFB: luxol fast blue
mAb: monoclonal antibody
MHC: major histocompatibility complex
MOG: myelin oligodendrocyte glycoprotein
MS: multiple sclerosis
PFA: paraformaldehyde
PMA: phorbol 12-myristate 13-acetate
TNF: tumor necrosis factor
Treg: regulatory T cell
WT: wild type

## References

[1] Lassmann H, van Horssen J, Mahad D (2012). Progressive multiple sclerosis: pathology and pathogenesis. Nat Rev Neurol..

[2] Gold R, Linington C, Lassmann H (2006). Understanding pathogenesis and therapy of multiple sclerosis via animal models: 70 years of merits and culprits in experimental autoimmune encephalomyelitis research. Brain..

[3] Rangachari M, Kuchroo VK (2013). Using EAE to better understand principles of immune function and autoimmune pathology. J Autoimmun..

[4] Fletcher JM, Lalor SJ, Sweeney CM, Tubridy N, Mills KH (2010). T cells in multiple sclerosis and experimental autoimmune encephalomyelitis. Clin Exp Immunol..

[5] Codarri L, Gyulveszi G, Tosevski V, Hesske L, Fontana A, Magnenat L (2011). RORgammat drives production of the cytokine GM-CSF in helper T cells, which is essential for the effector phase of autoimmune neuroinflammation. Nat Immunol..

[6] Ponomarev ED, Shriver LP, Maresz K, Pedras-Vasconcelos J, Verthelyi D, Dittel BN (2007). GM-CSF production by autoreactive T cells is required for the activation of microglial cells and the onset of experimental autoimmune encephalomyelitis. J Immunol..

[7] Comi G (2013). Disease-modifying treatments for progressive multiple sclerosis. Mult Scler..

[8] Simmons SB, Pierson ER, Lee SY, Goverman JM (2013). Modeling the heterogeneity of multiple sclerosis in animals. Trends Immunol..

[9] Hindinger C, Bergmann CC, Hinton DR, Phares TW, Parra GI, Hussain S (2012). IFN-gamma signaling to astrocytes protects from autoimmune mediated neurological disability. PLoS One..

[10] Smolen JS, Beaulieu A, Rubbert-Roth A, Ramos-Remus C, Rovensky J, Alecock E (2008). Effect of interleukin-6 receptor inhibition with tocilizumab in patients with rheumatoid arthritis (OPTION study): a double-blind, placebo-controlled, randomised trial. Lancet..

[11] Atreya R, Mudter J, Finotto S, Mullberg J, Jostock T, Wirtz S (2000). Blockade of interleukin 6 trans signaling suppresses T-cell resistance against apoptosis in chronic intestinal inflammation: evidence in Crohn’s disease and experimental colitis in vivo. Nat Med..

[12] Becker C, Fantini MC, Schramm C, Lehr HA, Wirtz S, Nikolaev A (2004). TGF-beta suppresses tumor progression in colon cancer by inhibition of IL-6 trans-signaling. Immunity..

[13] Rincon M, Irvin CG (2012). Role of IL-6 in asthma and other inflammatory pulmonary diseases. Int J Biol Sci..

[14] Kishimoto T, Akira S, Narazaki M, Taga T (1995). Interleukin-6 family of cytokines and gp130. Blood..

[15] Maimone D, Guazzi GC, Annunziata P (1997). IL-6 detection in multiple sclerosis brain. J Neurol Sci..

[16] Gijbels K, Van Damme J, Proost P, Put W, Carton H, Billiau A (1990). Interleukin 6 production in the central nervous system during experimental autoimmune encephalomyelitis. Eur J Immunol..

[17] Samoilova EB, Horton JL, Hilliard B, Liu TS, Chen Y (1998). IL-6-deficient mice are resistant to experimental autoimmune encephalomyelitis: roles of IL-6 in the activation and differentiation of autoreactive T cells. J Immunol..

[18] Okuda Y, Sakoda S, Bernard CC, Fujimura H, Saeki Y, Kishimoto T (1998). IL-6-deficient mice are resistant to the induction of experimental autoimmune encephalomyelitis provoked by myelin oligodendrocyte glycoprotein. Int Immunol..

[19] Mendel I, Katz A, Kozak N, Ben-Nun A, Revel M (1998). Interleukin-6 functions in autoimmune encephalomyelitis: a study in gene-targeted mice. Eur J Immunol..

[20] Dienz O, Rincon M (2009). The effects of IL-6 on CD4 T cell responses. Clin Immunol..

[21] Eugster HP, Frei K, Kopf M, Lassmann H, Fontana A (1998). IL-6-deficient mice resist myelin oligodendrocyte glycoprotein-induced autoimmune encephalomyelitis. Eur J Immunol..

[22] Bettelli E, Carrier Y, Gao W, Korn T, Strom TB, Oukka M (2006). Reciprocal developmental pathways for the generation of pathogenic effector TH17 and regulatory T cells. Nature..

[23] Korn T, Mitsdoerffer M, Croxford AL, Awasthi A, Dardalhon VA, Galileos G (2008). IL-6 controls Th17 immunity in vivo by inhibiting the conversion of conventional T cells into Foxp3+ regulatory T cells. Proc Natl Acad Sci U S A..

[24] Hirota H, Kiyama H, Kishimoto T, Taga T (1996). Accelerated nerve regeneration in mice by upregulated expression of interleukin (IL) 6 and IL-6 receptor after trauma. J Exp Med..

[25] Loddick SA, Turnbull AV, Rothwell NJ (1998). Cerebral interleukin-6 is neuroprotective during permanent focal cerebral ischemia in the rat. J Cereb Blood Flow Metab..

[26] Zhang PL, Izrael M, Ainbinder E, Ben-Simchon L, Chebath J, Revel M (2006). Increased myelinating capacity of embryonic stem cell derived oligodendrocyte precursors after treatment by interleukin-6/soluble interleukin-6 receptor fusion protein. Mol Cell Neurosci..

[27] Gruol DL, Nelson TE (1997). Physiological and pathological roles of interleukin-6 in the central nervous system. Mol Neurobiol..

[28] Erta M, Quintana A, Hidalgo J (2012). Interleukin-6, a major cytokine in the central nervous system. Int J Biol Sci..

[29] Brambilla R, Persaud T, Hu X, Karmally S, Shestopalov VI, Dvoriantchikova G (2009). Transgenic inhibition of astroglial NF-kappa B improves functional outcome in experimental autoimmune encephalomyelitis by suppressing chronic central nervous system inflammation. J Immunol..

[30] Brambilla R, Morton PD, Ashbaugh JJ, Karmally S, Lambertsen KL, Bethea JR (2014). Astrocytes play a key role in EAE pathophysiology by orchestrating in the CNS the inflammatory response of resident and peripheral immune cells and by suppressing remyelination. Glia..

[31] Campbell IL, Abraham CR, Masliah E, Kemper P, Inglis JD, Oldstone MB (1993). Neurologic disease induced in transgenic mice by cerebral overexpression of interleukin 6. Proc Natl Acad Sci U S A..

[32] Shih AY, Fernandes HB, Choi FY, Kozoriz MG, Liu Y, Li P (2006). Policing the police: astrocytes modulate microglial activation. J Neurosci..

[33] Starossom SC, Mascanfroni ID, Imitola J, Cao L, Raddassi K, Hernandez SF (2012). Galectin-1 deactivates classically activated microglia and protects from inflammation-induced neurodegeneration. Immunity..

[34] Hindinger C, Gonzalez JM, Bergmann CC, Fuss B, Hinton DR, Atkinson RD (2005). Astrocyte expression of a dominant-negative interferon-gamma receptor. J Neurosci Res..

[35] Chen Z, Jalabi W, Shpargel KB, Farabaugh KT, Dutta R, Yin X (2012). Lipopolysaccharide-induced microglial activation and neuroprotection against experimental brain injury is independent of hematogenous TLR4. J Neurosci..

[36] Wang J, Asensio VC, Campbell IL (2002). Cytokines and chemokines as mediators of protection and injury in the central nervous system assessed in transgenic mice. Curr Top Microbiol Immunol..

[37] Md Yusof MY, Emery P (2013). Targeting interleukin-6 in rheumatoid arthritis. Drugs..

[38] Serada S, Fujimoto M, Mihara M, Koike N, Ohsugi Y, Nomura S (2008). IL-6 blockade inhibits the induction of myelin antigen-specific Th17 cells and Th1 cells in experimental autoimmune encephalomyelitis. Proc Natl Acad Sci U S A..

[39] Basso AS, Frenkel D, Quintana FJ, Costa-Pinto FA, Petrovic-Stojkovic S, Puckett L (2008). Reversal of axonal loss and disability in a mouse model of progressive multiple sclerosis. J Clin Invest..

[40] Sofroniew MV, Vinters HV (2010). Astrocytes: biology and pathology. Acta Neuropathol..

[41] Black JA, Newcombe J, Waxman SG (2010). Astrocytes within multiple sclerosis lesions upregulate sodium channel Nav1.5. Brain..

[42] Okada S, Nakamura M, Mikami Y, Shimazaki T, Mihara M, Ohsugi Y (2004). Blockade of interleukin-6 receptor suppresses reactive astrogliosis and ameliorates functional recovery in experimental spinal cord injury. J Neurosci Res..

[43] Campbell IL, Erta M, Lim SL, Frausto R, May U, Rose-John S (2014). Trans-signaling is a dominant mechanism for the pathogenic actions of interleukin-6 in the brain. J Neurosci..

[44] Gimsa U, Mitchison NA, Brunner-Weinzierl MC (2013). Immune privilege as an intrinsic CNS property: astrocytes protect the CNS against T-cell-mediated neuroinflammation. Mediators Inflamm..

[45] Kimura A, Kishimoto T (2010). IL-6: regulator of Treg/Th17 balance. Eur J Immunol..

[46] Ajami B, Bennett JL, Krieger C, McNagny KM, Rossi FM (2011). Infiltrating monocytes trigger EAE progression, but do not contribute to the resident microglia pool. Nat Neurosci..

[47] Moreno M, Bannerman P, Ma J, Guo F, Miers L, Soulika AM (2014). Conditional ablation of astroglial CCL2 suppresses CNS accumulation of M1 macrophages and preserves axons in mice with MOG peptide EAE. J Neurosci..

[48] Streit WJ, Hurley SD, McGraw TS, Semple-Rowland SL (2000). Comparative evaluation of cytokine profiles and reactive gliosis supports a critical role for interleukin-6 in neuron-glia signaling during regeneration. J Neurosci Res..

[49] Min KJ, Yang MS, Kim SU, Jou I, Joe EH (2006). Astrocytes induce hemeoxygenase-1 expression in microglia: a feasible mechanism for preventing excessive brain inflammation. J Neurosci..

[50] Spooren A, Kolmus K, Laureys G, Clinckers R, De Keyser J, Haegeman G (2011). Interleukin-6, a mental cytokine. Brain Res Rev..

[51] Bruck W (2005). The pathology of multiple sclerosis is the result of focal inflammatory demyelination with axonal damage. J Neurol..

[52] Gijbels K, Brocke S, Abrams JS, Steinman L (1995). Administration of neutralizing antibodies to interleukin-6 (IL-6) reduces experimental autoimmune encephalomyelitis and is associated with elevated levels of IL-6 bioactivity in central nervous system and circulation. Mol Med..

[53] de Vries HE, Blom-Roosemalen MC, van Oosten M, de Boer AG, van Berkel TJ, Breimer DD (1996). The influence of cytokines on the integrity of the blood–brain barrier in vitro. J Neuroimmunol..

[54] Willenborg DO, Fordham SA, Cowden WB, Ramshaw IA (1995). Cytokines and murine autoimmune encephalomyelitis: inhibition or enhancement of disease with antibodies to select cytokines, or by delivery of exogenous cytokines using a recombinant vaccinia virus system. Scand J Immunol..

[55] Horwitz MS, Evans CF, Klier FG, Oldstone MB (1999). Detailed in vivo analysis of interferon-gamma induced major histocompatibility complex expression in the central nervous system: astrocytes fail to express major histocompatibility complex class I and II molecules. Lab Invest..

[56] Greter M, Heppner FL, Lemos MP, Odermatt BM, Goebels N, Laufer T (2005). Dendritic cells permit immune invasion of the CNS in an animal model of multiple sclerosis. Nat Med..

[57] McMahon EJ, Bailey SL, Castenada CV, Waldner H, Miller SD (2005). Epitope spreading initiates in the CNS in two mouse models of multiple sclerosis. Nat Med..

[58] Quintana A, Erta M, Ferrer B, Comes G, Giralt M, Hidalgo J (2013). Astrocyte-specific deficiency of interleukin-6 and its receptor reveal specific roles in survival, body weight and behavior. Brain Behav Immun..

[59] Haroon F, Drogemuller K, Handel U, Brunn A, Reinhold D, Nishanth G (2011). Gp130-dependent astrocytic survival is critical for the control of autoimmune central nervous system inflammation. J Immunol..

[60] Mishra PK, Hsuchou H, Ouyang S, Kastin AJ, Wu X, Pan W (2013). Loss of astrocytic leptin signaling worsens experimental autoimmune encephalomyelitis. Brain Behav Immun..

[61] Chiang CS, Stalder A, Samimi A, Campbell IL (1994). Reactive gliosis as a consequence of interleukin-6 expression in the brain: studies in transgenic mice. Dev Neurosci..

[62] Barnum SR, Jones JL, Muller-Ladner U, Samimi A, Campbell IL (1996). Chronic complement C3 gene expression in the CNS of transgenic mice with astrocyte-targeted interleukin-6 expression. Glia..

[63] Heyser CJ, Masliah E, Samimi A, Campbell IL, Gold LH (1997). Progressive decline in avoidance learning paralleled by inflammatory neurodegeneration in transgenic mice expressing interleukin 6 in the brain. Proc Natl Acad Sci U S A..

[64] Molnarfi N, Schulze-Topphoff U, Weber MS, Patarroyo JC, Prod’homme T, Varrin-Doyer M (2013). MHC class II-dependent B cell APC function is required for induction of CNS autoimmunity independent of myelin-specific antibodies. J Exp Med..

[65] Barr TA, Shen P, Brown S, Lampropoulou V, Roch T, Lawrie S (2012). B cell depletion therapy ameliorates autoimmune disease through ablation of IL-6-producing B cells. J Exp Med..

